# Oxidative stress modulation in type 2 diabetes: Insights from metformin and pioglitazone therapy

**DOI:** 10.5937/jomb0-59850

**Published:** 2025-09-05

**Authors:** Seshadri Reddy Varikasuvu

**Affiliations:** 1 All India Institute of Medical Sciences (AIIMS), Department of Biochemistry, Deoghar, Jharkhand, India

**Keywords:** oxidative stress, type 2 diabetes mellitus, metformin, pioglitazone, oksidativni stres, dijabetes melitus tipa 2, metformin, pioglitazon

## Abstract

This commentary critically examines a recent study that assessed the effects of metformin and pioglitazone on oxidative stress in patients with type 2 diabetes mellitus. The original study utilized multiple biomarkers such as IMA, TOS, TAS, and OSI but observed only limited antioxidant effects and a rise in IMA levels. By referencing additional clinical trials, experimental studies, and reviews, this commentary emphasizes the importance of using broader biomarker panels, extending treatment duration, and interpreting oxidative stress markers with caution in diabetes research.

## Abstract (sr)

Ovaj komentar kritički ispituje nedavnu studiju koja je procenila efekte metformina i pioglitazona na oksidativni stres kod pacijenata sa dijabetesom melitusom tipa 2. Originalna studija je koristila više biomarkera kao što su IMA, TOS, TAS i OSI, ali je primetila samo ograničene antioksidativne efekte i porast nivoa IMA. Pozivajući se na dodatna klinička ispitivanja, eksperimentalne studije i preglede, ovaj komentar naglašava važnost korišćenja širih panela biomarkera, produženja trajanja lečenja i tumačenja markera oksidativnog stresa sa oprezom u istraživanju dijabetesa.

## 55

*Dear Editor,*

We read with considerable interest the article by Temiz Genço lu et al. titled »Effects of Use of Metformin or Combination of Metformin and Pioglitazone on Oxidative Stress in Type 2 Diabetes Mellitus« [1]. The study addresses an important and timely topic, as oxidative stress is increasingly recognized as a contributor to both the development and progression of diabetic complications.

A key strength of the study lies in its inclusion of multiple biomarkers—Ischemia-Modified Albumin (IMA), Total Oxidant Status (TOS), Total Antioxidant Status (TAS), and the Oxidative Stress Index (OSI). This comprehensive approach is well-suited to capture the complexity of oxidative balance. The authors reported a statistically significant reduction in TOS and OSI in the group receiving metformin and pioglitazone, though no significant differences were observed between the treatment arms for final biomarker values. An unexpected rise in IMA levels was noted in both groups. While the study is a welcome contribution, some limitations deserve consideration. A 12-week intervention may be insufficient to observe sustained changes in oxidative parameters, particularly those involved in chronic metabolic regulation. Previous research has shown more pronounced antioxidant effects with longer treatment durations. For instance, Balamurugan et al. reported reductions in oxidative and inflammatory markers after 24 weeks of pioglitazone treatment in patients with type 2 diabetes [2].

The scope of biomarkers used, though broad, did not include lipid peroxidation products or enzymatic antioxidants such as malondialdehyde (MDA), superoxide dismutase (SOD), or glutathione peroxidase. These markers are frequently used in oxidative stress research and could provide further insight. Singh et al. reported significant changes in MDA and SOD with both metformin and pioglitazone after only four weeks of treatment [3]. Additional support for the antioxidant role of these agents comes from a randomized controlled trial by Mirmiranpour et al., which compared the effects of metformin and pioglitazone on markers such as advanced oxidation protein products, advanced glycation end products, and enzymatic antioxidants. Both agents significantly improved oxidative stress profiles, with each showing unique effects on specific parameters [4]. These findings are reinforced by experimental evidence. In diabetic rat models, combination therapy with metformin and pioglitazone led to marked reductions in oxidative damage markers and improvements in antioxidant enzyme levels [5]. Similar improvements in mitochondrial function and hepatic oxidative balance have been documented in diabetic mice treated with the same combination [6]. A review by Rizvi and Mishra concluded that metformin consistently reduced several oxidative stress markers across multiple studies [7]. Yet in the study by Temiz Genço lu et al., metformin monotherapy did not significantly reduce TOS or OSI levels. This variation may be attributed to differences in patient characteristics, biomarker selection, or treatment duration.

Human studies on the antioxidant effects of metformin and pioglitazone have shown variable results. While some clinical trials have demonstrated improvements in oxidative and inflammatory markers with either agent, others highlight inconsistency depending on patient characteristics, baseline metabolic control, or biomarker selection. For instance, Hu et al. reported improved oxidative profiles following metformin therapy, particularly in individuals with poor glycaemic control [8], whereas Bulatova et al. observed that metformin combined with lifestyle modification reduced oxidative markers significantly, while lifestyle intervention alone paradoxically increased them [9]. These discrepancies underscore the complexity of oxidative stress in clinical settings. Notably, the increase in IMA observed in both treatment arms in the current study further raises concerns about its specificity and utility. Given that IMA is known to be influenced by ischemia, inflammation, and albumin-binding alterations, it may not reliably reflect oxidative stress in isolation, especially without accompanying clinical or biochemical correlates [8] [9] [10] [11].

Conclusively, the work by Temiz Genço lu et al. contributes meaningful data to the study of oxidative stress in diabetes management. Future studies would benefit from extended follow-up periods, inclusion of additional oxidative biomarkers, and assessment of relevant clinical outcomes. These enhancements could offer a more definitive picture of how antidiabetic therapies modulate oxidative stress and related complications.

## Dodatak

### Acknowledgements

Dr. S.R. Varikasuvu specially acknowledges Bhairavi Sisters (Sahasra and Aagneya) for the time he could not give them during the preparation of this letter.

### Funding

The author(s) received no financial support for the research, authorship, and/or publication of this article.

### Ethical Committee Approval

Not applicable, because this article does not contain information from studies using human or animal subjects.

### Declaration of Conflicting Interests

The author(s) declared no potential conflicts of interest with respect to the research, authorship, and/or publication of this article.
